# Assessing the effect of elevated CO_2_ on wheat leaf rust severity: insights from a free-air carbon dioxide enrichment (FACE) experiment

**DOI:** 10.3389/fpls.2026.1939751

**Published:** 2026-10-16

**Authors:** Jasper Krößmann, Lars Großhans-Kretschmer, Lisa Waßmann, Bernd Rodemann, Lorenz Kottmann, Sven E. Weber, Rod J. Snowdon, Albrecht Serfling, Andreas Stahl

**Affiliations:** 1 Julius Kühn-Institute (JKI) – Federal Research Centre for Cultivated Plants, Institute for Resistance Research and Stress Tolerance, Quedlinburg, Germany; 2 Julius Kühn-Institute (JKI) – Federal Research Centre for Cultivated Plants, Institute for Crop and Soil Science, Braunschweig, Germany; 3 Julius Kühn-Institute (JKI) – Federal Research Centre for Cultivated Plants, Institute for Field Crops and Grasland Protection, Braunschweig, Germany; 4 Department of Plant Breeding, Justus Liebig University, Giessen, Germany

**Keywords:** climate adaptation, elevated carbon dioxide, leaf rust, phenotyping, winter wheat

## Abstract

**Introduction:**

The continuous increase in atmospheric carbon dioxide (CO_2_) concentrations is a major driver of global climate change and may alter plant growth and host–pathogen interactions. Understanding how elevated CO_2_ affects disease development is therefore important for future crop production and resistance screening. This study investigated the effect of elevated CO_2_ on wheat leaf rust (*Puccinia triticina*) in Central European winter wheat genotypes under field conditions.

**Methods:**

Free Air Carbon Dioxide Enrichment (FACE) field experiments were conducted over two growing seasons, exposing winter wheat to an atmospheric CO_2_ concentration of approximately 600 ppm. Leaf rust development was assessed using the Area Under the Disease Progress Curve (AUDPC) and Average Ordinate (AO). Genotypic effects, CO_2_ treatment effects and their interaction were analysed across the tested genotypes and growing seasons.

**Results:**

Elevated CO_2_ was associated with a general tendency towards higher leaf rust severity, although the magnitude of this response varied between growing seasons and genotypes. Genotype effects were highly significant, whereas the genotype × CO_2_ interaction was comparatively weak, providing limited evidence for differential responses among the tested genotypes. Under the conditions of this FACE experiment, the relative ranking of genotypes for leaf rust severity was largely maintained under elevated CO_2_.

**Discussion:**

The results indicate that elevated CO_2_ may affect the expression of wheat leaf rust resistance, while the relative differences among genotypes remained largely consistent under the tested conditions. However, the limited number of independent CO_2_ treatment environments, particularly following the exclusion of one elevated CO_2_ ring in 2023, restricts the strength of treatment-level inference. The findings should therefore be interpreted as observations specific to the experimental conditions rather than as general evidence for stable leaf rust resistance under elevated atmospheric CO_2_. Nevertheless, they highlight the importance of considering changing atmospheric CO_2_ concentrations when evaluating and screening disease resistance in future wheat breeding programmes.

## Highlights

- Elevated CO_2_ (~600 ppm) slightly increased wheat leaf rust severity
- Genotypic effects were highly significant, while CO_2_ × genotype interaction was weak
- Relative resistance rankings of winter wheat genotypes remained stable under elevated CO_2_
- Disease assessment using AUDPC and AO captured consistent CO_2_-driven trends across two years

## 1 Introduction

The concentration of atmospheric carbon dioxide (CO_2_) has increased from approximately 280 parts per million (ppm) in pre-industrial times to over 420 ppm today, and this increase is projected to continue (Meinshausen et al., 2020; NOAA, 2025). This increase is one of the primary drivers of climate change, because CO_2_ significantly impacts global temperatures by intensifying the greenhouse effect (Ainsworth and Rogers, 2007). Climate change poses a significant challenge, to agriculture, affecting both crop productivity and susceptibility to pathogens (Miedaner and Juroszek, 2021).

Wheat (*Triticum* spp.) is one of the most important staple crops for human consumption. In 2023, it was cultivated on approximately 220 million hectares globally (FAO, 2025). Studies indicate that rising CO_2_ levels can positively impact on C3 plants such as wheat, by increasing photosynthetic performance, enhancing biomass production, and enlarging leaf area (Ainsworth and Rogers, 2007; Fitzgerald et al., 2016). Higher productivity can result from increased photosynthetic efficiency, particularly through improved CO_2_ fixation and enhanced water and nutrient uptake (Ewert et al., 2002; Ainsworth and Rogers, 2007). However, these effects vary depending on the wheat variety and growing region, necessitating differentiated adaptation strategies (Hasegawa et al., 2013; Tausz-Posch et al., 2015; Sanz-Sáez et al., 2017). Conversely, a larger leaf area could increase the surface area vulnerable to pathogen attack (Chakraborty et al., 2011).

The interaction between wheat growth and pathogens is influenced by several factors. In addition to increased leaf area, changes in the microclimatic conditions within the crop stand may also affect infection rates (Soriano and Royo, 2015; Caubel et al., 2017; Wójtowicz et al., 2017; Miedaner and Juroszek, 2021). Studies further suggest that smaller stomata, reduced transpiration rates, and altered wax layers could hinder pathogen penetration (Eastburn et al., 2011), which could lead to increased resistance.

One of the most significant wheat pathogens is leaf rust (*Puccinia triticina*), an obligate biotrophic fungus that requires intact leaf tissue to develop (Bancal et al., 2007). Infection by this pathogen can result in yield losses of up to 60% (Bancal et al., 2007; Kolmer, 2013). Previous research on leaf rust resistance has primarily focused on race-specific resistances and pyramidization. Particular attention has been given to genes that mediate specific defense responses against certain rust races (Zetzsche et al., 2019; Beukert et al., 2020). However, the impact of rising atmospheric CO_2_ concentrations and climatic changes have largely been neglected. The current state of research on the interaction between increasing CO_2_ levels and foliar pathogen development is inconsistent and is based on only a limited number of genotypes (Tiedemann and Firsching, 2000; Melloy et al., 2010; Chakraborty et al., 2011; Gullino et al., 2018; Solanki et al., 2019). To our knowledge, no study has examined the effects of atmospheric CO_2_ levels on infection and resistance behavior across a wider range of wheat genotypes.

Practical assessments of the effects of elevated atmospheric CO_2_ concentrations on crops and pathogens can only be achieved through field trials using Free-Air Carbon dioxide Enrichment (FACE) technology. In FACE experiments, CO_2_ levels are controlled directly in the field, enabling plants to grow outside of closed chambers. This enables a more realistic simulation of future atmospheric conditions (Ainsworth and Rogers, 2007; Kimball, 2016) To our knowledge, FACE experiments on wheat in combination with studies on an obligate biotrophic pathogen have only been conducted once for stripe rust, revealing no significant impact of elevated CO_2_ concentrations (Chakraborty et al., 2011).

In this study, we hypothesize that i) elevated atmospheric CO_2_ concentration increases leaf rust infection and ii) not all genotypes respond to changes in CO_2_ concentrations in the same way, resulting in a genotype-CO_2_ concentration interaction for leaf rust severity. The aim of this study is to investigate the impact of elevated CO_2_ concentrations on the severity of Central European winter wheat to the leaf rust pathogen through two-year FACE field experiments.

## 2 Materials and methods

### 2.1 Plant material

This study focuses on twelve different genotypes of winter wheat from Central Europe. The genotypes were selected according to the following criteria: variability in agronomic traits, quality, year of registration, leaf rust resistance ratings, and economic relevance. A detailed list of genotype-specific information can be found in **Supplementary Table 1**.

### 2.2 Experimental design of the FACE experiments

The field experiments were conducted in Braunschweig (52.29° N, 10.44° E), Northern Germany, in the years 2023 and 2024. Each year, four FACE rings and four corresponding ambient control rings, which did not undergo CO_2_ enrichment, were used. One FACE ring and one ambient ring were paired with each other, resulting in four ring pairs in each experimental year. Investigations of leaf rust were conducted on only two of these ring pairs in each year (**Figure 1**). The two remaining ring pairs were not inoculated with leaf rust and are therefore not included in this study. In each ring, the twelve varieties were cultivated in duplicate in a fully randomised design (**Supplementary Figure S1**). The plot size was always 3.75 m² (3 m × 1.25 m). The detailed functionality and logistics of the FACE system, including the daily enrichment of CO_2_ to a target concentration of 600 ppm from sunrise to sunset, starting at spring emergence and continuing until harvest, are described in Kottmann et al. (2025). According to Kottmann et al. (2025), the seasonal mean CO_2_ concentration reached 600 ppm in both 2023 and 2024. Fumigation was active for 93% of the intended fumigation time in 2023 and 87% in 2024, with interruptions caused by high wind speeds and, in 2024, an additional gas tank cooling system malfunction.

**Figure 1:**
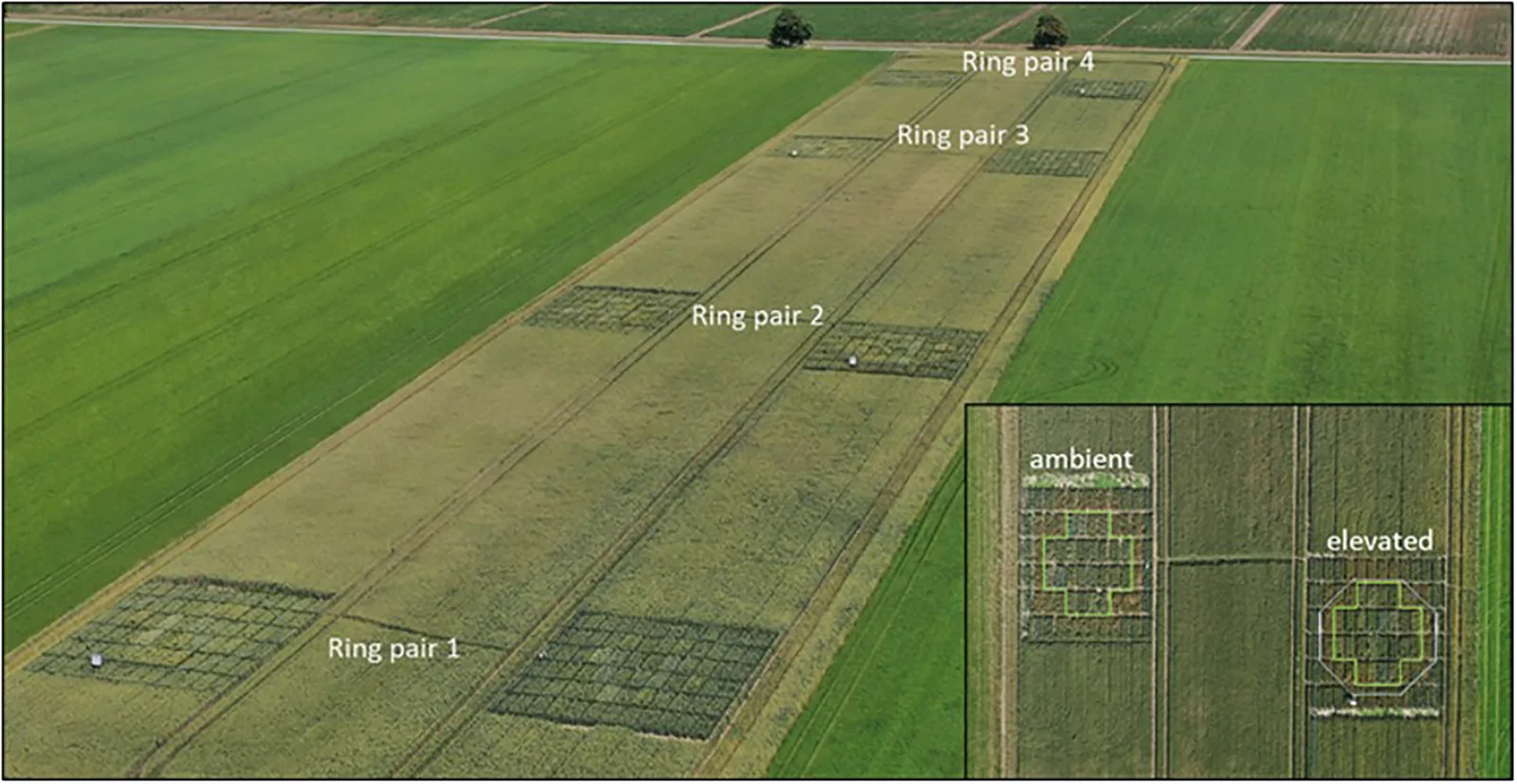
Drone images of the complete experimental field showing all four ring pairs. Leaf rust experiments were conducted in both experimental years using ring pairs 2 and 4. Inset at the bottom right: image of an ambient control ring and a FACE elevated ring (ring pair). Green-marked areas indicate the respective test plots within each ring. Brownish discoloration is visible in the outer sections at the infection plots of the cultivar *Borenos*, caused by artificially induced leaf rust infection.

### 2.3 Artificial inoculation and phenotypic assessment of leaf rust resistance

To ensure a uniform leaf rust infection in the experimental plots, the leaf rust-susceptible standard cv. *Borenos* was cultivated as an infection host around the relevant experimental rings for leaf rust investigations (**Figure 1**; **Supplementary Figure S1**). At the onset of heading (Zadoks growth stage 50, Zadoks et al., 1974), the infection hosts were artificially inoculated with the highly virulent *Puccinia triticina* isolate "77WxR" (Zetzsche et al., 2019). A spore suspension containing 10 mg of uredospores in 100 ml of Isopar M (ExxonMobil Chemical Company, USA) was applied at a rate of 10 ml per m² using a hand-held spinning disc sprayer (Bromyard, U.K.) (Rollar et al., 2021). The isolate was propagated in a greenhouse at 17 °C and nearly 90% humidity on 20 seedlings of the cv. *Borenos*, which were grown in pots with a diameter of 15 cm (Beukert et al., 2021).

Phenotyping of the test plots was carried out by visually assessing the percentage of infected leaf area on the flag leaves and assigning a representative disease severity value to each test plot. The flag leaves of the plants within each plot were considered collectively, without a predefined number of plants or flag leaves or a predefined selection procedure, and a single estimated percentage of infected leaf area was assigned to the plot. Thus, no plot-level mean was calculated from individual plant or leaf ratings. At each assessment date, the respective plot was assessed as a whole, and individual plants or flag leaves were not followed over time. Assessments were conducted at the same defined days post-inoculation (dpi) for all genotypes within each year. All disease assessments in both experimental years were performed by the same person to ensure consistency of scoring. The assessor was aware of the genotype and CO_2_ treatment during the assessments. In the 2023 field experiment, three assessments were conducted at 22, 37, and 51 days post-inoculation (dpi), whereas in 2024, four assessments were performed at seven-day intervals, starting at 19 dpi and continuing until 40 dpi. Genotype-specific phenological data were not recorded. Consequently, phenological differences among genotypes and potential effects of elevated CO_2_ on phenological development could not be evaluated or accounted for analytically.

At physiological maturity (Zadoks growth stage 89; Zadoks et al., 1974), each plot was harvested. A 0.5 m² sub-area was hand-harvested, while the remaining plot area (3.25 m²) was harvested using a plot combine (Hege 160, Hans-Ulrich Hege Saatzuchtmaschinen, Hohebuch, Germany). The hand-harvested grain was separated from straw and additionally threshed using a laboratory thresher (LD 180, Wintersteiger, Ried, Austria). Grain yield (GY, g m^-^²) was calculated from the combined hand- and machine-harvested grain fractions and standardised to 14% moisture content.

### 2.4 Statistical analysis

The area under the disease progress curve (AUDPC) and the average ordinate (AO) were calculated for each variety based on multiple ratings of the percentage of infected leaf area (Moll et al., 1996) according to Equations 1 and 2:

(1) $$AUDPC=\sum_{i=1}^{N_{i-1}}\frac{\left(y_{i}+y_{i+1}\right)}{2}\times \left(t_{i+1}-t_{i}\right)$$

and

(2) $$AO=\frac{AUDPC}{T}$$

where *y_i_* is the disease level at the *i*-th observation, *t_i_* is the time at the *i*-th observation, *N* is the total number of observations and *T* is the total observation duration, measured in days from the first to the last rating date (Rollar et al., 2021).

Variance component analyses were conducted on a ring-specific and year-specific basis to determine the variance components of the AUDPC values at the end of the season attributable to variety and positional effects (Equation 3). Additionally, Equation 4 was applied to the two-year combined data across rings to estimate variance components associated with variety, treatment, year, and their respective interaction effects. All variance component analysis equations were performed using the ‘lme4’ package (Bates et al., 2015).

(3) $$P_{ijk}=\mu +{\text{G}}_{i}+{\text{R}}_{j}+{\text{C}}_{k}+e_{ijk}$$

where *P_ijk_* represents the phenotypic value of the *i*-th genotype, the *j*-th row and the *k*-th column. *µ* is the general mean. G*_i_* is the random effect of the *i*-th genotype, R*_j_* is the random effect of the *j*-th row and C*_k_* is the random effect of the *k*-th column. *e_ijk_* is the random error term.

(4) $$P_{ijkl}=\mu +{\text{G}}_{i}+{\text{T}}_{j}+{\text{Y}}_{k}+{\text{Rp}}_{l}+{\left(\text{YT}\right)}_{kj}+{\left(\text{YG}\right)}_{ki}+{\left(\text{TG}\right)}_{ji}+{\left(\text{YTG}\right)}_{kji}+e_{ijkl}$$

where *P_ijkl_* represents the phenotypic value for the *i*-th genotype, the *j*-th treatment, the *k*-th year and the *l*-th Ring pair. *μ* is the overall mean, G*_i_* represents the random effect of the *i*-th genotype, T*_j_* the random effect of the *j*-th treatment, Y*_k_* the random effect of the *k*-th year and Rp*_l_* the random effect of the *l*-th ring pair. The random interaction effects are as follows: (YT)*_kj_* for year × treatment, (YG)*_ki_* for year × genotype, (TG)*_ji_* for treatment × genotype and (YTG)*_kji_* for year × treatment x genotype. *e_ijkl_* is the random error term.

Adjusted means, (analysis of variance) ANOVA, and Fisher’s least significant difference (LSD) test (*p* < 0.05) were calculated for all traits measured. The single-year data from 2023 and 2024 were analyzed separately using Equation 5, while the analysis across both years were conducted using Equation 6 via the ‘lme4’ package (Bates et al., 2015):

(5) $$P_{ijl}=\mu +{\text{g}}_{i}*{\text{t}}_{j}+{\text{rp}}_{l}+{\left(\text{RpT}\right)}_{lj}+{\left(\text{RpTG}\right)}_{lji}+e_{ijl}$$

where *P_ijl_* represents the phenotypic value for the *i*-th genotype, the *j*-th treatment and the *l*-th ring pair. *μ* is the overall mean, g*_i_* represents the fixed effect of the *i*-th genotype, t*_j_*​ the fixed effect of the *j*-th treatment, respectively, including their two-and three-way interactions. rp*_l_* represents the fixed effect of the *l*-th ring pair. (RpT)*_lj_* for ring pair x treatment and (RpTG)*jkm​* for ring pair x treatment x genotype. *e_ijl_* is the random error term.

(6) $$P_{ijkl}=\mu +{\text{g}}_{i}*{\text{t}}_{j}*{\text{y}}_{k}+{\text{rp}}_{l}+{\left(\text{YRpT}\right)}_{klj}+{\left(\text{YRpTG}\right)}_{klji}+e_{ijkl}$$

where *P_ijkl_* represents the phenotypic value for the *i*-th genotype, the *j*-th treatment, the *k*-th year and the *l*-th Ring pair. *μ* is the overall mean, g*_i_* represents the fixed effect of the *i*-th genotype, t*_j_* the fixed effect of the *j*-th treatment, y*_k_* represents the fixed effect of the *k*-th year, respectively, including their two-and three-way interactions. rp*_l_* represents the fixed effect of the *l*-th ring pair. (YRpT)*_klj_* is the random effect of the year x ring pair x treatment interaction, while (YRpTG)_*klji*_ is the random effect for year x ring pair x treatment x genotype interaction. *e_ijkl_* is the random error term.

The broad-sense heritability across years and CO_2_ treatments for each individual trait was estimated following the approach described by Piepho and Möhring (2007); Equation 19. To this end, the model specified in Equation 6 was fitted as a fully random model in order to obtain estimates of the genotypic variance component. The standard error of the difference between two entry means was derived using the same model, except that genotype was fitted as a fixed effect.

(7) $$h^{2}=\frac{\sigma_{g}^{2}}{\sigma_{g\;}^{2}+\frac{SE^{2}}{2}}$$

where $\sigma_{g}^{2}$ donates the genetic variance estimated from the full random model (Equation 6). SE^2^ denotes the squared standard error of the difference between the means estimated using the model specified in Equation 6, with genotype treated as a fixed effect and all other factors retained as random effects.

For the estimation of heritability within a single CO_2_ treatment across both years, as well as within a single year across both CO_2_ treatments, the same procedure was applied. However, in these cases the respective factor (treatment or year) was omitted from the model, and the calculations were performed on the corresponding subset of the dataset.

All analyses were performed using R v4.1.2 (Team R.C, 2020). In addition to the aforementioned packages, we used the ‘ggplot2’ package (Wickham, 2016) for data visualisation. The Pearson coefficient of correlation was calculated based on adjusted means and plots were created using the ‘corrplot’ R package (Wei et al., 2021). The significance levels of AUDPC and AO were calculated for genotypes, varieties and treatments. The calculation of the significance levels of AUDPC and AO for genotypes, varieties and treatments was performed using t-test.

## 3 Results

Ring-specific variance component analyses revealed that the factor ‘*genotype*’ consistently accounted for the largest proportion of variance (usually over 90%), while contributions from positions within the rings from residual components were only marginally. An exception was FACE Ring 2 in year 2023, which showed an unusually high residual deviation of 37.53%. The full random model was subsequently used to assess how the proportions of the key experimental factors changed depending on whether the ring was retained in the study or excluded due to its unexplained high residual variance. The results showed that the effects remained similar in magnitude for the overall study, while the adjusted means for both genotype and CO_2_ remained largely unchanged. Consequently, this ring was excluded from the data analyses to ensure data integrity (**Supplementary Figure S3**).

The disease variables were designated as Disease Severity 1 (DS1), Disease Severity 2 (DS2), Disease Severity 3 (DS3), and, only in 2024, Disease Severity 4 (DS4), representing disease severity recorded at the first, second, third, and fourth assessment dates, respectively. In contrast, AUDPC was calculated from disease severity values across multiple assessment dates and represents the integrated development of leaf rust over the respective assessment period. AUDPC was calculated separately for 2023 and 2024 and for the combined data across both years.

Variance components for the trait AUDPC using two-year combined data across rings revealed that the effect of genotype (G) accounted for by far the largest proportion of the total variance, clearly emerging as the dominant source of variation. The second-largest contribution originated from the genotype × year (G × Y) effect, although its magnitude remained substantially smaller than that of the main genotype effect. All remaining interaction terms contributed only marginally to the overall variance. Likewise, the residual component represented only a small proportion compared with the genetic effects and their year-dependent expression. Overall, these results show that genetic differences, together with their modulation across years, predominantly shape the variability for AUDPC, whereas all other sources of variation play only a subordinate role (**Supplementary Figure S3**). In 2023, disease-related traits showed an increase from DS1 to the integrated AUDPC value under both ambient and elevated CO_2_ conditions. Mean values ranged from 0.94 (DS1) to 226.1 (AUDPC) under ambient conditions, with high repeatabilities of up to 0.99. For all examined traits, elevated CO_2_ treatments resulted in higher values overall, with AUDPC showing a representative value of 350.9. In 2024, a similar trend was observed. Under ambient CO_2_ treatment, means ranged from 5.5 (DS1) to 230.9 (AUDPC), whereas under elevated CO_2_ conditions, mean values ranged from 6.9 to 258.3. Maximum disease severity values (DS1, DS3, and DS4) and AUDPC were again higher under elevated CO_2_ conditions. Only the DS2 maximum value was higher under ambient CO_2_ treatment. Repeatabilities followed a similar pattern to the previous year and were marginally higher under ambient conditions (up to *h*^2^ = 0.98) than under elevated CO_2_ conditions (up to *h*^2^ = 0.94). A two-year analysis confirmed higher minimum, maximum, and mean values for all traits under elevated CO_2_ conditions. Heritability values remained similar across CO_2_ conditions, with slightly higher values under ambient CO_2_ conditions (**Table 1**). Differences in leaf rust infection progression were also evident in the average ordinate (AO). In 2023, the average AO was 7.79% under ambient CO_2_ conditions and 12.1% under elevated CO_2_ conditions. In 2024, slightly higher mean values were observed, reaching 10.99% and 12.29%, respectively. The two-year analysis confirmed these trends and differences. Additionally, the findings indicated that repeatabilities and heritability were lower under elevated CO_2_ conditions than under ambient CO_2_ conditions. Distinct differences in yield emerged between treatments and years. In 2023, mean yield was higher than in 2024, with a lower repeatability in 2023 under ambient CO_2_ (*h*^2^ = 0.30) and markedly higher in 2024 (*h*^2^ = 0.91). Over two years, the average yield was 1065 g/m² under elevated CO_2_ conditions and 779.1 g/m² under ambient CO_2_ conditions. Minimum and maximum yield values followed the same trend. Heritability values were slightly higher under ambient CO_2_ conditions (*h*^2^ = 0.60) than under elevated CO_2_ conditions (*h*^2^ = 0.50) (**Table 1**).

**Table 1:** Descriptive statistics and least significant differences calculated according to Equations 5 and 6, and repeatability/heritability estimates calculated according to Equation 7 for the investigated traits.

Year	Trait^a^	Unit	Ambient					Elevated					LSD^g^
			Min^b^	Max^c^	Mean	*rep/h^2^*	n^f^	Min^b^	Max^c^	Mean	*rep/h^2^*	n^f^	
2023	DS1	–	0	6.75	0.94	0.90^d^	48	0.19	9.19	3.75	–	24	5.21
	DS2	–	18.75	367.5	78.6	0.99^d^	48	26.1	416.1	124.22	–	24	75.93
	DS3	–	35	665	146.56	0.99^d^	48	50.9	750.9	222.9	–	24	150.98
	AUDPC	–	53.8	1039	226.1	0.99^d^	48	77.2	1176	350.9	–	24	240.36
2024	DS1	–	0	26.3	5.5	0.94^d^	48	0	33.3	6.9	0.59^d^	48	12.89
	DS2	–	2.63	118.1	26.3	0.97^d^	48	2.63	116.4	29.24	0.81^d^	48	29.65
	DS3	–	6.1	231.9	62.1	0.98^d^	48	11.4	240.6	72.41	0.94^d^	48	42.32
	DS4	–	17.5	315	136.9	0.98^d^	48	28	332.5	149.7	0.92^d^	48	117.27
	AUDPC	–	26.3	691.3	230.9	0.98^d^	48	42	722.8	258.3	0.93^d^	48	169.05
Both years	DS1	–	0	16.5	3.24	0.59^e^	96	0.31	21.44	5.55	0.57^e^	72	8.59
	DS2	–	12.44	242.8	52.46	0.71^e^	96	21.8	272	82.5	0.53^e^	72	38.6
	DS3	–	25.81	448.4	104.3	0.74^e^	96	45.5	506.2	158.13	0.58^e^	72	70.65
	AUDPC	–	50.75	865.3	228.5	0.92^e^	96	100	970.7	325.86	0.78^e^	72	162.66
2023	AO	%	1.85	35.84	7.79	0.99^d^	48	2.66	40.56	12.1	–	24	7.47
2024	AO	%	1.25	32.92	10.99	0.98^d^	48	2	34.42	12.29	0.93^d^	48	8.05
Both years	AO	%	1.92	34.38	9.39	0.96^e^	96	4.14	38.37	13.08	0.84^e^	72	6.97
2023	Yield	g/m^2^	704	958	839	0.30^d^	48	1167	1565	1396	–	24	447.79
2024	Yield	g/m^2^	531.1	862.4	718.4	0.91^d^	48	589	1035	802.11	0.94^d^	48	108.52
Both years	Yield	g/m^2^	639.43	900.8	779.1	0.60^e^	96	886	1205	1065	0.50^e^	72	222.9

^a^ DS1, DS2, DS3, DS4: Disease Severity values recorded at the first to fourth scoring time points during the vegetation period, respectively; AUDPC_o: cumulative area under the disease progress curve calculated over the entire assessment period; AO: average ordinate calculated from AUDPC_o and the assessment duration.

^b^ Minimum

^c^ Maximum

^d^ Repeatability (rep)

^e^ Heritability (*h*^2^)

^f^ number of samples

^g^ Least significant difference

In 2023, the genotype effect was statistically significant for all DS and AUDPC traits (*p* < 0.001). The CO_2_ treatment effect was not significant for any of the traits. In contrast, the interaction effect between genotype and treatment (G × T) was significant for all traits, with (*p* < 0.01) for DS1 and (*p* < 0.001) for all other traits. In 2024, the genotype effect remained highly significant for all DS and AUDPC traits (*p* < 0.001). Neither the CO_2_ concentration effect nor the interaction effect was statistically significant for any of the assessed traits. A significant effect of ring pair was only detected for DS2 in 2024 (*p* < 0.01). Across both experimental years, the genotype effect was consistently highly significant (*p* < 0.001). The treatment effect was not statistically significant for any of the assessed traits. The interaction effect was not statistically significant for DS1, but for the other traits DS2 and DS3 (*p* < 0.001) and *p*<0.01 for AUDPC. The genotype effect for the AO values was consistently highly significant (*p* < 0.001). The treatment effect was not statistically significant in either year. The interaction effect was highly significant in 2023 (*p* < 0.001), though somewhat weaker across both years (*p* < 0.05). For yield, the genotype effect was non-significant in 2023, whereas it was significant in 2024 and in the combined two-year analysis (*p* < 0.001). Here, the treatment effect was significant in both years, with *p* < 0.01 in 2023 and *p* < 0.001 in 2024, whereas the interaction effect was not statistically significant in either year. A significant year effect was detected only for DS1–DS3, with p<0.01 for DS1 and p<0.05 for both DS2 and DS3, whereas no significant year effect was observed for AUDPC (**Table 2**).

**Table 2:** *p*-values from ANOVA tests for the investigated traits according to Equations 5 and 6.

Year	Trait^a^	Unit	Genotype	CO_2_ Treatment	Ring pair	Year	GxT^b^	GxY^c^	TxY^d^	GxTxY^e^
2023	DS1	–	<0.001	0.213	0.867	–	<0.01	–	–	–
	DS2	–	<0.001	0.166	0.486	–	<0.001	–	–	–
	DS3	–	<0.001	0.253	0.565	–	<0.001	–	–	–
	AUDPC	–	<0.001	0.249	0.574	–	<0.001	–	–	–
2024	DS1	–	<0.001	0.448	0.356	–	0.997	–	–	–
	DS2	–	<0.001	0.487	<0.01	–	0.965	–	–	–
	DS3	–	<0.001	0.477	0.237	–	0.961	–	–	–
	DS4	–	<0.001	0.726	0.487	–	0.503	–	–	–
	AUDPC	–	<0.001	0.640	0.365	–	0.895	–	–	–
Both years	DS1	–	<0.001	0.062	0.307	<0.01	0.989	<0.01	0.443	0.997
	DS2	–	<0.001	0.125	0.984	<0.05	<0.001	<0.001	0.148	<0.001
	DS3	–	<0.001	0.130	0.885	<0.05	<0.001	<0.001	0.182	<0.001
	AUDPC	–	<0.001	0.187	0.669	0.317	<0.01	<0.001	0.291	<0.001
2023	AO	%	<0.001	0.184	0.516	–	<0.001	–	–	–
2024	AO	%	<0.001	0.640	0.365	–	0.895	–	–	–
Both years	AO	%	<0.001	0.225	0.571	0.736	<0.05	<0.001	0.378	<0.05
2023	Yield	g/m^2^	0.092	<0.01	0.441	–	0.163	–	–	–
2024	Yield	g/m^2^	<0.001	<0.001	<0.01	–	0.367	–	–	–
Both years	Yield	g/m^2^	<0.001	0.052	0.799	0.042	0.275	<0.001	0.096	<0.01

^a^ DS1, DS2, DS3, DS4: Disease Severity values recorded at the first to fourth scoring time points during the vegetation period, respectively; AUDPC: cumulative area under the disease progress curve calculated over the entire assessment period; AO: average ordinate calculated from AUDPC and the assessment duration.

^b^ Genotype and CO_2_ treatment interaction

^c^ Genotype and year interaction

^d^ CO_2_ treatment and year interaction

^e^ Genotype and treatment and year three-way interaction

For non-significant effects, exact p-values are reported, whereas significant effects are indicated by significance levels: * for p<0.05, ** for p<0.01, and *** for p<0.001.

The differing leaf rust severity levels of the investigated wheat genotypes were consistently confirmed within the framework of the two-year analysis of genotype-specific traits for both the AUDPC and the AO. Notably, the genotype cultivar 6 exhibited the highest leaf rust severity under both CO_2_ conditions, with statistically significant differences compared to most other genotypes. In contrast, the genotypes cultivar 3 and cultivar 7 displayed the lowest leaf rust severity, although this difference was not always statistically significant across all comparisons.

A detailed trait-specific analysis revealed that all examined genotypes exhibited increased AUDPC values under elevated CO_2_ conditions. However, this increase was statistically significant only for the genotypes cultivar 4 and 10, suggesting that these genotypes may be particularly sensitive to altered atmospheric CO_2_ concentrations. A similar trend was observed for AO, where an overall increase under elevated CO_2_ conditions was evident (**Figure 2**).

**Figure 2:**
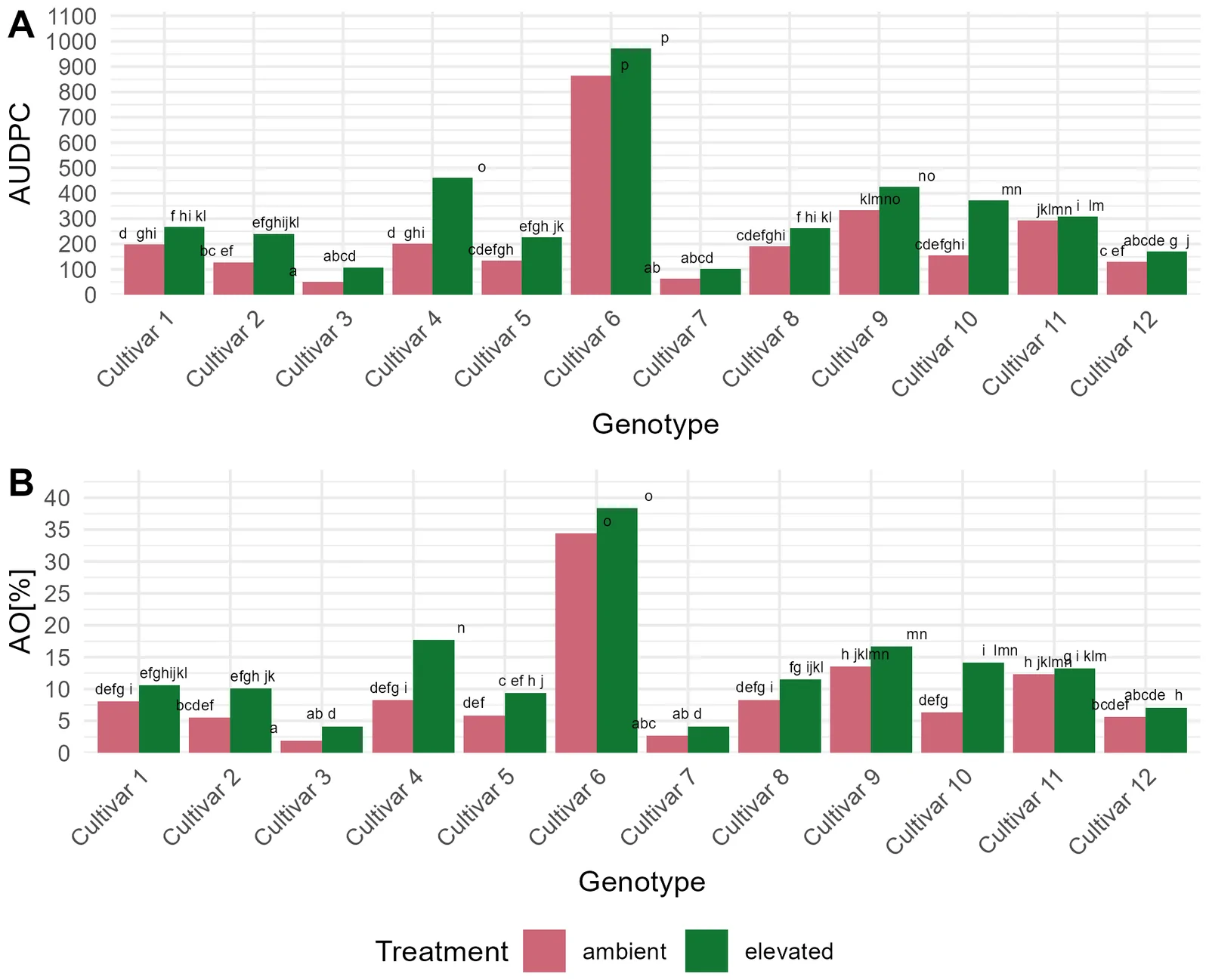
Genotype-specific trait expressions based on two-year analysis for **(A)** AUDPC values and **(B)** AO values. Shown data are adjusted means according to Equation 6. Significances (*p* < 0.05) between varieties and treatments were tested with t-test.

To contextualise the experimental years, disease severity and infection parameters were analyzed comparatively. Overall, higher levels of disease incidence and infection were observed in year 2023 compared with 2024. Significant cultivar differences were detected only in 2023 for both, AUDPC and AO, for the cultivars 4, 9 and 10 exhibiting significantly higher leaf rust severity under elevated CO_2_ conditions (**Supplementary Figures S4**, **S5**).

These findings suggest that elevated atmospheric CO_2_ concentrations may generally exacerbate disease progression, although the extent of this effect varies considerably between genotypes. The observed differences among genotypes highlight the complex interactions between host and pathogen under changing climatic conditions and emphasize the necessity of targeted breeding strategies that consider environmental and genetic factors influencing disease severity into account.

The results of the variance analyses for the individual years, as well as the two-year overall analysis, demonstrated that differences in the examined traits between the two treatments were rarely significant and, if so, only marginally. To further support these observations, year-specific correlation analyses were conducted for the individual traits.

In both experimental years, leaf rust severity traits showed strong positive correlations between ambient and elevated CO_2_ conditions, with some correlations reaching very high coefficients. In 2024 in particular, the correlations were stronger than in 2023, reaching values of up to r = 0.98 (**Figure 3**). These results indicate a high degree of consistency in the relative ranking of genotypes between CO_2_ conditions. The two-year analysis also revealed high Pearson correlations, although these were generally slightly lower than those observed in the individual year analyses. Substantial differences in correlation values were observed between the two years for yield, with r = 0.08 in 2023 and r = 0.92 in 2024. Together with the differences observed between 2023 and 2024, these results indicate that the two years differed in their environmental conditions, which may have affected trait expression and genotype responses. The two-year analysis further showed that differences in annual environmental conditions could not be fully neutralised, suggesting that both leaf rust severity and yield were influenced by the specific growing conditions of each year. The correlation analyses therefore indicate a certain stability in the relative ranking of genotypes between CO_2_ conditions, while also revealing considerable interannual variation. The stronger correlations observed in 2024 compared with 2023 suggest greater consistency in genotype ranking between CO_2_ conditions in 2024. In contrast, the lower correlations in 2023 indicate greater variation in genotype responses between the two CO_2_ conditions.

**Figure 3:**
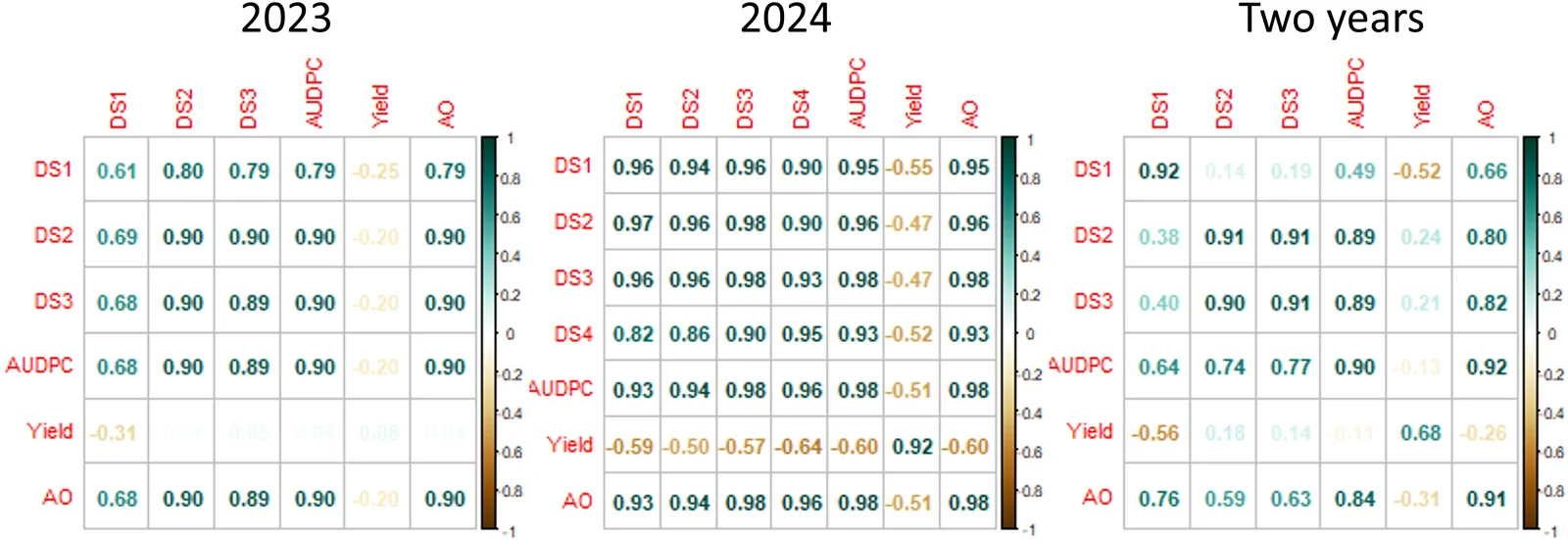
Pearson correlation coefficients between adjusted genotype means under ambient and elevated CO_2_ conditions for the investigated traits. Correlations are based on twelve genotypes and are shown separately for the individual experimental years and the combined two-year analysis. Pearson correlation coefficients and corresponding p-values are provided for all pairwise correlations. Significant correlations (*p* < 0.05) are shown as filled ellipses, whereas non-significant correlations remain unfilled. Green indicates positive correlations, whereas yellow indicates negative correlations. The intensity of the colors reflects the strength of the correlations, as shown by the scale beside each plot. The triangular structure avoids redundant presentation of the same pairwise correlations.

## 4 Discussion

The two-year FACE experiments demonstrate that elevated atmospheric CO_2_ concentrations can affect the development of leaf rust in winter wheat. Overall, CO_2_ enrichment resulted in higher disease pressure, as indicated by increased AUDPC and AO values. However, the extent of this increase varied between years and genotypes. Therefore, the results suggest that, although elevated CO_2_ concentrations may promote stronger disease development, the magnitude of this effect depends heavily on genetic and environmental factors.

### 4.1 Effect of elevated CO_2_ concentrations on disease development

The observed trend towards higher leaf rust severity under elevated CO_2_ is consistent with the hypothesis that elevated CO_2_ concentrations may indirectly create microclimatic conditions favorable to pathogen development (Miedaner and Juroszek, 2021). CO_2_-induced increases in photosynthetic performance, biomass production, and leaf area have been suggested to contribute to denser canopies, resulting in higher relative humidity and reduced air movement within crop stands in the field (Knopf et al., 2023). Such microclimatic changes may favor biotrophic fungi such as leaf rust (Chakraborty et al., 2011; Eastburn et al., 2011). In the present study, these potential microclimatic differences between elevated and ambient CO_2_ conditions may therefore have contributed to the observed differences in disease development.

However, the different assessment periods between the two experimental years also need to be considered when interpreting disease development. Because the timing and duration of disease assessments differed between years, raw AUDPC values are not directly comparable across years. Therefore, AO, which standardises AUDPC for the duration of the assessment period, provides a more suitable measure for comparing overall disease development between years. The individual disease severity values (DS1–DS4) provide additional information on disease severity at specific assessment time points and allow disease progression to be characterised over the course of the assessment period. These differences in assessment periods further emphasise the need for cautious interpretation of interannual differences in disease development.

A further limitation of the present study is that genotype-specific phenological development was not recorded during the disease assessments. Consequently, potential differences among genotypes in heading, flag-leaf emergence, senescence, or green-leaf duration, as well as potential effects of elevated CO_2_ on phenological development, could not be quantified or accounted for analytically. Disease assessments were conducted at defined days post-inoculation and based on a representative visual estimate of infected flag-leaf area at the plot level. This provided a consistent assessment of disease severity across genotypes and experimental years, but does not exclude the possibility that differences in developmental stage contributed to variation in apparent leaf rust severity. The results should therefore be interpreted with this limitation in mind. Future experiments should record genotype- and treatment-specific phenology alongside disease development to allow potential phenological effects on disease severity to be evaluated.

Physiologically, elevated CO_2_ may affect several processes relevant to rust infection, including processes associated with pathogen penetration through the stomata. Previous research has shown that elevated atmospheric CO_2_ can reduce stomatal density (Franks et al., 2013), potentially influencing gas exchange and leaf moisture. Furthermore, an altered carbon–nitrogen balance may affect nutrient availability for pathogens. An excess of carbon and a relative nitrogen deficiency may lead to thicker tissue structures and modified defense responses, which can have positive or negative effects depending on the pathogen (Miedaner and Juroszek, 2021).

The increase in disease severity observed under elevated CO_2_ in some genotypes may therefore be partly explained by indirect physiological and microclimatic effects on the host plant. However, these mechanisms were not directly measured in the present study and should therefore be regarded as possible explanations rather than demonstrated mechanisms. Similarly, the high repeatability and heritability estimates observed in this study should be interpreted in the context of the selected genotype set and the limited number of independent treatment environments. These estimates primarily reflect the reliability of genotype differences under the specific experimental conditions rather than heritability in a broader breeding population.

### 4.2 Genotypic differences but weak interactions with CO_2_

Analysis of the genotypes revealed significant differences in leaf rust severity, whereas the genotype × CO_2_ concentration interaction was relatively weak. This suggests that the genotypes responded broadly similarly to elevated CO_2_, despite an increase in absolute disease severity. Accordingly, the relative ranking of genotype responses appeared largely stable under the CO_2_ conditions tested in the present experiments. This is a relevant observation for resistance breeding, as it suggests that current resistance assessments may remain informative under elevated CO_2_ conditions (Miedaner and Juroszek, 2021; Knopf et al., 2023; Smith and Luna, 2023).

However, the limited number of independent CO_2_ treatment replicates restricts the statistical power to detect treatment and genotype-specific responses. Although two ambient and two elevated-CO_2_ rings were available for the leaf rust experiments in each year, the 2023 data analysis included only one elevated-CO_2_ ring because the second elevated ring was excluded due to heterogeneity. Thus, the treatment effect was based on two independent ambient and one independent elevated-CO_2_ ring in 2023, and on two independent ambient and two independent elevated-CO_2_ rings in 2024. These limited numbers of independent treatment environments, together with the exclusion of one elevated-CO_2_ ring in 2023, require cautious interpretation of the treatment effects and the genotype × CO_2_ interaction. In particular, the absence of a strong genotype × CO_2_ interaction should not be taken as evidence that resistance rankings or disease responses will remain stable under all future CO_2_ scenarios. Accordingly, the observed treatment effects should be interpreted as differences in leaf rust severity under the specific experimental conditions rather than as evidence of a general CO_2_ effect on disease susceptibility.

The variation in the magnitude of the response among individual genotypes nevertheless suggests that physiological or structural traits may contribute to differences in disease development under elevated CO_2_. For example, differences in plant growth and canopy structure could alter the canopy microclimate and thereby affect conditions conducive to leaf rust development. Studies on wheat growth under elevated CO_2_ have shown that CO_2_ enrichment can enhance photosystem II efficiency and vegetative biomass (Knopf et al., 2023), which may indirectly influence canopy conditions. However, these physiological and microclimatic mechanisms were not directly measured in the present study and should therefore be regarded as possible explanations rather than demonstrated mechanisms.

### 4.3 Molecular – physiological context

The results of this study are consistent with the current literature describing heterogeneous effects of elevated CO_2_ on plant diseases. The present study provides further field-based evidence of these effects in a set of twelve winter wheat genotypes. While Chakraborty et al. (2011) and Eastburn et al. (2011) found no significant CO_2_ effects for other wheat biotrophic pathogens (like rusts), Solanki et al. (2019) reported increased disease susceptibility in other plant species. These discrepancies may be related to differences among pathosystems, climatic conditions, host genotypes and experimental setups, including greenhouse and field-based studies.

Recent studies indicate that elevated CO_2_ can influence hormone signaling pathways that are relevant to plant defense. For example, elevated CO_2_ may enhance salicylic acid (SA)-dependent defense against biotrophic pathogens while simultaneously weakening jasmonic acid (JA)-dependent defense against necrotrophic pathogens (Smith and Luna, 2023).

However, since leaf rust is a biotrophic fungus, enhanced SA-mediated defense responses may have had compensatory effects, which could explain why the observed CO_2_ effectremained moderate in this study. Contradictory findings in the literature highlight the complexity of CO_2_–plant–pathogen interactions: elevated CO_2_ does not act directly on the pathogen but rather alters plant physiology, microclimate, and defense balance— positive and negative effects that overlap (Smith and Luna, 2023). According to this study, as a positive effect in a part of investigated wheat genotypes, higher yield and biomass could be detected, independent from higher infection values under elevated.

### 4.4 Methodological aspects and future research

Thus, this study contributes to the growing body of evidence that rising CO_2_ represents both an opportunity and a risk for plant health: it can enhance growth and resource-use efficiency, while at the same time subtly altering disease dynamics – a challenge for sustainable breeding and protection strategies under climate change. The results are based on two years of experiments conducted at a single site, which is a common limitation of FACE experiments (Kottmann et al., 2025). Interannual variations in temperature, humidity, and precipitation can strongly influence disease development (Knopf et al., 2023). To robustly quantify CO_2_ concentration effects, multi-location and multi-year studies, integrating both physiological and molecular variables are therefore required.

Future research should particularly aim to combine disease quantification with (i) measurements of stomatal density and leaf nutrient content (Franks et al., 2013), (ii) analyses of defense hormone profiles (Smith and Luna, 2023), and (iii) assessments of microclimatic parameters within the canopy.

This integrated approach would provide allow for a more comprehensive understanding of the physiological and environmental mechanisms underlying the observed CO_2_ effects on disease development. This would enable the empirical validation of the proposed mechanisms and their incorporation into breeding and plant protection models.

## 5 Conclusion

The present FACE results indicate that elevated CO_2_ concentrations were associated with increased leaf rust severity in winter wheat, while the relative ranking of the genotypes remained largely stable. The observed differences in disease development may be associated with indirect effects of elevated CO_2_ on plant physiology and the crop microclimate, but these mechanisms were not directly assessed in the present study. As the study was conducted at a single site over two years, further multi-year and multi-site studies are needed to assess the consistency of these responses and to evaluate the combined effects of CO_2_, temperature, and precipitation on plant–pathogen interactions and disease development.

## Data Availability

The raw data supporting the conclusions of this article will be made available by the authors, without undue reservation.
