# Supplementary material for: Assessing the effect of elevated CO2 on wheat leaf rust severity: insights from a free-air carbon dioxide enrichment (FACE) experiment
Source: Front Plant Sci. 2026 Oct 16;17:1939751. doi: 10.3389/fpls.2026.1939751 (PMC13721790; doi:10.3389/fpls.2026.1939751)
Supplement: Supplementary file 1 [file DataSheet1.docx]

**Assessing the Effect of Elevated CO₂ on Wheat Leaf Rust Infection: Insights from a Free-Air Carbon Dioxide Enrichment (FACE) Experiment**

Jasper Krößmann, Lars Großhans-Kretschmer, Lisa Wassmann, Bernd Rodemann, Lorenz Kottmann, Sven E. Weber, Rod J. Snowdon, Albrecht Serfling and Andreas Stahl

**Supplementary Information**

**Supplementary Table 1:** **Overview of the wheat genotypes included in this study and their years of release.**

| Number | Genotype | Year of Release |
| --- | --- | --- |
| 1 | Anapolis | 2013 |
| 2 | Benchmark | 2015 |
| 3 | Informer | 2018 |
| 4 | JB Asano | 2008 |
| 5 | Kerubino | 2004 |
| 6 | KWS Donovan | 2020 |
| 7 | RGT Reform | 2014 |
| 8 | SU Selke | 2019 |
| 9 | SU Willem | 2022 |
| 10 | SW Topper | 2002 |
| 11 | Tobak | 2011 |
| 12 | Toras | 2004 |

**Supplementary Figure 1: Example of the methodology employed in cultivating the leaf rust susceptible standard cv. *Borenos* as an infection host around the experimental plots.**

**Supplementary Figure 2: Proportions of variance explained by different components for the trait AUDPC for individual experimental rings in both experimental years. Variance components for these factors were calculated based on the model described in equation 3. Panels represent the following experimental setups: (A) Ambient ring 1 in 2023; (B) Elevated ring 1 in 2023; (C) Ambient ring 2 in 2023; (D) Elevated ring 2 in 2023; (E) Ambient ring 1 in 2024; (F) Elevated ring 1 in 2024; (G) Ambient ring 2 in 2024; (H) Elevated ring 2 in 2024.**

**Supplementary Figure 3: Proportions of variance components for the trait AUDPC based on two-year combined data, quantifying the contributions of variety, treatment, year, and their respective interaction effects according to Equation 4. (A) All rings included in both years; (B) Ring 2 from 2023 excluded.**

**Supplementary Figure 4: Genotype-specific trait expressions for the 2023 experimental year showing (A) AUDPC values and (B) AO values. Values represent adjusted means calculated according to Equation 5. Differences between varieties and treatments were assessed using Tukey’s test (*p* < 0.05).**

**Supplementary Figure 5: Genotype-specific trait expressions for the 2024 experimental year showing (A) AUDPC values and (B) AO values. Values represent adjusted means calculated according to Equation 5. Differences between varieties and treatments were assessed using Tukey’s test (*p* < 0.05).**
